# Safety and efficacy of ultrasound-guided distal forearm median nerve block for pediatric trigger thumb release: a retrospective observational study in a southern Chinese cohort

**DOI:** 10.3389/fmed.2025.1648300

**Published:** 2025-09-16

**Authors:** Xiang-Xuan Wang, Wen-Chen Xu, Tuan-Fang Fang, Zi-Hong Zhao, Kai-Nan Lin, Hui Chen

**Affiliations:** ^1^Department of Pediatric Orthopedics, Fujian Children’s Hospital (Fujian Branch of Shanghai Children’s Medical Center), College of Clinical Medicine for Obstetrics and Gynecology and Pediatrics, Fujian Medical University, Fuzhou, China; ^2^Department of Anesthesiology, Fujian Children's Hospital (Fujian Branch of Shanghai Children’s Medical Center), College of Clinical Medicine for Obstetrics and Gynecology and Pediatrics, Fujian Medical University, Fuzhou, China; ^3^Graduate College of Fujian Medical University, Fuzhou, China

**Keywords:** interventional ultrasonography, nerve block, trigger thumb disorder, pediatrics, study, retrospective

## Abstract

**Purpose:**

The aim of this study was to evaluate the clinical outcomes of ultrasound-guided distal forearm median nerve block (UDFMNB) in children undergoing A1 pulley release.

**Methods:**

We conducted a retrospective analysis of the clinical data of unilateral pediatric trigger thumb (PTT) patients who underwent A1 pulley release under UDFMNB with American Society of Anesthesiologists (ASA) status I at a large pediatric hospital in South China from June 2021 to December 2024. Patients were divided into two groups based on age (≤3 years and >3 years). The data collected included sex, affected side, age at onset, age at surgery, preoperative symptoms, Sugimoto staging, surgical time, operating room stay duration, block success rate, preoperative and postoperative range of motion of the thumb IP joint, and final follow-up time. Successful UDFMNB was defined as a complete sensory block without the need for additional analgesics or conversion to general anesthesia. Pain intensity was assessed using an 11-point numerical rating scale (NRS), and the satisfaction of surgeons and parents of patients with anesthesia was evaluated using a 7-point Likert scale. Complications were also recorded.

**Results:**

This study involved 65 patients (32 boys and 33 girls) with a median age at surgery of 1.8 years. The affected sides were left (*n* = 29) and right (*n* = 36), with 63 patients presenting with IP joint flexion contracture and 2 with extension. Using the Sugimoto staging system, the cases of 12 patients were classified as stage III and the cases of 53 patients were classified as stage IV. After stratification by age, 21 and 44 patients were included in the two groups. The overall success rate of UDFMNB was 93.8%, with a higher failure rate in the ≤3-year group than in the >3-year group (4.62% vs. 1.54%). The operative duration was 15 (14–23) min, with no surgery-related complications. Two patients (one per group) experienced transient puncture-site redness. All patients regained thumb mobility without reoperation. The median NRS pain scores were 2 intraoperatively and 1 postoperatively, with no intergroup differences.

**Conclusion:**

Satisfaction scores were high among surgeons and parents of patients. In conclusion, UDFMNB has high success rates and an excellent safety profile for analgesia in pediatric trigger thumb release surgery, making it suitable for clinical application and wider adoption.

## Introduction

Pediatric trigger thumb (PTT) is one of the most common hand conditions, accounting for approximately 2% of all upper limb deformities in children (1, 2). The incidence rate is approximately 0.5 to 3 per 1,000 children (2). PTT is characterized by flexion deformity of the interphalangeal joint (IP) (3), and a small number of cases are characterized by extension fixation deformity (4).

The impaired performance of the IP joint is attributed to the mismatch between the thumb A1 pulley and the flexor pollicis longus (FPL) tendon on the affected side (5), leading to abnormal tendon sliding. The FPL tendon is stimulated and thickened, and finally, the palpable palmar nodules form at the metacarpophalangeal joint (MCP), known as Notta’s node (6).

A1 pulley release is the recommended treatment for trigger thumb in children (7–10). The operation is often performed under general anesthesia. Although general anesthesia is widely used, it requires large doses of other drugs, increasing concerns about the risks to the neurodevelopment of young children; however, it is unclear whether it has a significant effect on the immature brain and whether a safer alternative is needed (11). To reduce bleeding in the operation area, a tourniquet is often used in the forearm. Pediatric patients are often unable to tolerate local anesthesia due to fear of surgery and unbearable discomfort caused by tourniquets.

In recent years, studies have reported that ultrasound-guided distal forearm median nerve block (UDFMNB) has achieved good results in children receiving hand and wrist surgery (12). Its advantages include a short onset time of anesthesia and retention of finger and proximal muscle motor function (13); at present, the effect of this technique in PTT surgery is still unclear. Since June 2021, we have attempted to treat PTT with A1 pulley release under UDFMNB. The purpose of this study was to evaluate the clinical effect of A1 pulley release under UDFMNB in the treatment of PTT and to evaluate its effectiveness and safety. We hypothesized that ultrasound-guided distal forearm median nerve block in pediatric trigger thumb surgery would have a high success rate and desirable outcomes.

## Materials and methods

### Study design and patients

The study was approved and supervised by the Ethics Committee of Fujian Children’s Hospital (2025ETKLRK08003) and was conducted in accordance with the ethical standards set out in the 1964 Declaration of Helsinki. Additionally, all parents of the children included in the study provided written informed consent after being fully informed about the study procedures, including surgical methods, preoperative medication, sedation, and forearm distal median nerve block.

### Data collection

We conducted retrospective data extraction from our institutional electronic medical records system for patients with trigger thumb treated between June 2021 and December 2024.

### Inclusion and exclusion criteria

The inclusion criteria were as follows:

1. Patients diagnosed with unilateral trigger thumb requiring surgery under ultrasound-guided distal peripheral median nerve block anesthesia.
2. Those with an ASA physical status of 1.
3. Those with postoperative follow-up for 3 months or longer.
4. Those whose legal guardians approved the treatment plan and provided informed consent.

The exclusion criteria were as follows:

1. Patients with juvenile arthritis, mucopolysaccharidosis, or a history of previous surgery on the affected thumb.
2. Those with infection at the puncture site.
3. Those with pre-existing peripheral neuropathy, chronic pain syndrome, congenital diabetes, allergy to study medications, or coagulation disorders.
4. Those with incomplete follow-up data.

The surgical indications for trigger thumb include patients older than 1 year with fixed trigger thumb flexion or extension locking for more than 6 months. All patients who received surgical treatment were subjected to A1 pulley release and partial resection of the affected thumb’s cutting pulley. To be included in this study, we selected patients with PTT who received a minimum of 3 months of follow-up after surgery. Only patients who met the aforementioned inclusion and exclusion criteria were enrolled in this study. All surgeries were performed by two pediatric orthopedic surgeons, and UDFMNB was performed by one of two senior anesthesiologists with >5 years of experience in ultrasound-guided pediatric nerve blocks.

Successful UDFMNB was defined as a complete sensory block without the need for additional analgesics or conversion to general anesthesia. The children were divided into two groups according to their age (≤3 years and >3 years). Data collection included information on sex, age at onset, age at surgery, affected side, preoperative symptoms, Sugimoto staging, operation time, operating room stay duration, block success rate, preoperative and postoperative IP joint range of motion of the thumb, and time to final follow-up. The pain intensity of the children was evaluated, and the satisfaction of the surgeons and the families of patients with anesthesia and the occurrence of complications were recorded.

### Preoperative management

After a specialist diagnosed the trigger thumb condition, patients were evaluated for surgical indications. Those with surgical indications were admitted to the hospital for preoperative assessments and preparations, excluding any contraindications for surgery. For patients with unilateral thumb involvement, the decision to use UDFMNB for anesthesia was made by the anesthesiologist after a comprehensive preoperative evaluation.

Preoperative fasting guidelines were followed (6 h for solid foods, 4 h for formula milk, and 2 h for clear liquids). First, 30 min before the start of anesthesia, lidocaine gel was locally applied to the skin at the injection site. Preoperative administration of dexmedetomidine nasal drops was determined on the basis of the patient’s clinical condition (Dosage and Administration: The typical administered dose is 1–1.5 μg/kg, with a single maximum dose not exceeding 50 μg. It should be administered approximately 30 min prior to surgery. The patient should be placed in a sitting or head-tilted-back position. Using a specialized nasal delivery device or syringe, the solution is instilled slowly into the nasal cavity, with care taken to avoid causing choking or coughing. After instillation, the patient should maintain the head-tilted-back position for 1–2 min to facilitate drug absorption) (14). Then, 20 min later, standard cardiopulmonary monitoring [electrocardiogram (ECG), noninvasive blood pressure (BP), heart rate (HR), respiratory rate (RR), mean arterial pressure (MAP), and pulse oximetry test (SpO_2_)] was initiated, and vascular access was established in the contralateral arm. If necessary, 1–2 mg/kg propofol was administered for light sedation before surgery and maintained at 5 mg/kg/h during the procedure.

### Ultrasound-guided distal forearm median nerve block

In accordance with the study conducted by Jalil et al. (15), a distal forearm median nerve block was performed. The patient was placed in a supine position with the affected limb abducted and externally rotated on the operating table, the palm facing upward, and the wrist slightly extended to fully expose the distal forearm. The proposed puncture area was disinfected routinely and draped. A high-frequency linear array probe equipped with a sterile probe cover was used to perform a baseline examination of the proposed puncture site. The puncture site and ultrasound transducer were aseptically prepared using a General Electric ultrasound machine equipped with a high-frequency linear transducer (GE L4-12T-RS, 4.2–12.0 MHz), and the ultrasound probe was placed on the distal forearm (2–3 cm proximal to the wrist crease) and tilted slightly toward the distal end to locate the median nerve (which appears as an oval-shaped hyperechoic structure on the fascial plane between the deep and superficial flexors).

**Figure 1:**
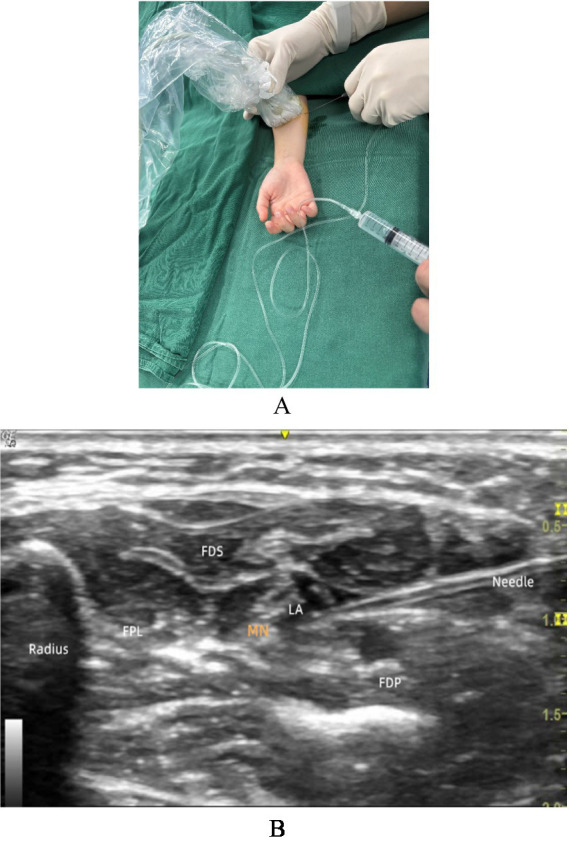
Localization of the median nerve. **(A)** UDFMNB operation diagram; **(B)** UDFMNB ultrasound imaging. MN, median nerve; FPL, flexor pollicis longus; FDS, flexor digitorum superficialis; FDP, flexor digitorum profundus; LA, local anesthesia.

A 22G short bevel needle was inserted from the radial side of the probe, with the needle tip parallel to the long axis of the probe. Using the intramuscular technique, the needle is inserted from the ulnar side to the radial side. The needle body and tip were visible under ultrasound throughout the procedure. The needle tip was slowly advanced to the periphery of the median nerve (under the neurilemma or within the fascial space), avoiding direct puncture of the nerve. A small amount of local anesthetic was injected, and the spread of the medication around the nerve was observed (ideally, the medication should encircle the nerve, presenting a “Halo sign”). If the spread was inadequate, the needle tip position was adjusted before reinjection. 0.2% ropivacaine was provided under ultrasound guidance until the median nerve was surrounded by local anesthetic, with a maximum volume lower than 0.5 mL/kg, and the expansion of the perineural space during injection was observed to avoid excessive pressure (Due to its lower efficacy in sensorimotor blockade and reduced cardiotoxicity, ropivacaine is routinely used at our center. In cases where ropivacaine is unavailable, alternative local anesthetics such as levobupivacaine may be selected based on the hospital’s drug availability) (Figure 1).

### Intraoperative evaluation and surgical protocol

Anesthesia was maintained by administering propofol injections as needed during the operation. If the child woke up or had unconscious body movement unrelated to the operation, an additional dose of 1 mg/kg propofol was provided until the child fell asleep again or the activity stopped. If there was a withdrawal reflex associated with pain stimulation, 0.1 μg/kg sufentanil was provided until the reflex was suspended. If the block failed and the surgeon could not continue the operation, the anesthesiologist, on the basis of experience, provided sufficient general anesthetics to meet the needs of the operation. If respiratory depression was observed or SpO_2_ was less than 95%, artificial open airway and pure oxygen mask pressure ventilation were performed immediately, and laryngeal mask or tracheal intubation was performed if necessary.

After the completion of nerve block, the surgeon assessed the effect of the median nerve block by pinching the skin at the surgical site with forceps before surgery. This assessment was conducted every 2 min to evaluate the onset time of anesthesia. Complete anesthesia was defined as a full (grade 1) or partial motor block (grade 2) without pain (complete sensory block to pinprick). Incomplete anesthesia was characterized by partial motor block with mild pain, requiring additional intravenous analgesics (grade 3), or incomplete motor block with severe pain, necessitating general anesthesia (grade 4) (16).

HR, RR, MAP, and SpO_2_ were continuously monitored during the operation, and five time points were recorded: anesthesia induction (Ta), skin incision (Tb), and skin closure (Tc). If the patient met any of the following criteria, the block was considered unsuccessful: (I) withdrawal reflex associated with surgical stimulation; (II) two or more additional doses of propofol or sufentanil were required during surgery; (III) the HR or MAP increased by more than 20% after the start of surgery; or (IV) severe pain was felt immediately after recovery from anesthesia. Intraoperative or postoperative adverse events, such as urinary retention, itching, nausea and vomiting, respiratory depression and tracheal spasm, were recorded. Intraoperative pain was assessed using the 11-point numerical rating scale (NRS) (17).

All patients underwent surgical release for trigger thumb. A transverse incision was made after a tourniquet was applied to the distal forearm. Following the incision of the skin and subcutaneous tissues and clear separation of the neurovascular bundle, the A1 pulley was longitudinally released, and a portion of the thickened tendon sheath was incised and excised. Passive motion of the interphalangeal joint was performed to confirm the effectiveness of the release. The skin was closed with absorbable sutures, and a soft dressing was applied with pressure to cover the wound. The thumb interphalangeal joint was immobilized in extension with a bandage, allowing the child to freely move the thumb within the dressing. Postoperative follow-up was scheduled and guided for all patients.

### Postoperative management

Postoperatively, all patients were observed in the recovery room for 1–2 h before being transferred to the general ward. During their hospital stay, patients who experienced intolerable postoperative pain received ibuprofen via the rectal or oral route for analgesic treatment. The NRS was used to assess patients’ pain immediately after surgery and on the first postoperative day. On the first postoperative day, a 7-point Likert scale (18) was used to evaluate the satisfaction of both doctors and patients’ families with perioperative analgesia. Patients were discharged after being evaluated by surgeons and anesthesiologists as meeting the discharge criteria. Before discharge, parents were provided with training and guidance on postoperative care for the affected limb and the management of anesthesia-related adverse reactions (19). Postoperative observation was conducted to monitor for any adverse reactions to anesthesia, such as nausea and vomiting, as well as complications, including inflammation or infection at the anesthesia and surgical site, wound healing issues, recurrence, thumb pain, numbness, and bowstring deformity. Active exercises of the metacarpophalangeal joint of the affected thumb and wrist joint were encouraged in pediatric patients when clinically permissible. These measures further reduced the risk of local adhesion formation at the puncture site.

### Statistical analysis

Statistical analyses were performed using SPSS 22.0 (IBM Corp, Armonk, NY, USA). Initially, all the data were screened for completeness, consistency, and outliers. Continuous variables are expressed as the means ± standard deviations with ranges, whereas categorical variables are represented as frequencies (%) or as median (IQR). Due to the non-normal distribution of the data, the nonparametric Mann–Whitney U test or Kruskal–Wallis test was employed to compare continuous variables among subgroups, and proportions were compared using the chi-square test. A *p*-value of <0.05 was considered statistically significant.

## Results

This study ultimately included 65 patients, comprising 32 boys and 33 girls, with 29 cases affecting the left side and 36 affecting the right side. The age at onset was 1.6 (1.2–4.9) years, while the age at surgery was 1.9 (1.8–5.5) years. Among them, 63 patients were presented with fixed IP joint flexion contracture and two with fixed IP joint extension. According to Sugimoto staging, 12 patients had stage III cases and 53 had stage IV cases. The patients were divided into two groups based on age: the under 3-year-old group (n = 21) and the 3-year-and-older group (n = 44). All patients were followed up for at least 3 months postoperatively. The detailed demographic characteristics and intergroup comparisons are presented in Table 1.

**Table 1:** Demographic data of the patients included in the study.

Item	Total *n* = 65	≤3 year *n* = 21	>3 year *n* = 44	*p* value
Sex (girls/boys)	33/32	9/12	24/20	0.544
Weight (kg)	11.2 ± 4.2	12.5 ± 3.6	16.1 ± 2.3	0.385
Side (left/right)	29/35	10/11	19/25	0.421
Age at onset (year)	1.6 (1.2–4.9)	1.3 (1.2–2.6)	3.2 (3.0–4.9)	0.348
Age at surgery (year)	1.9 (1.8–5.8)	2.1 (1.8–3.2)	3.6 (3.2–5.8)	0.455
IP fixed flexion	63 (96.9%)	20 (95.2%)	43 (97.7%)	0.234
IP extension	2 (3.1%)	1 (4.8%)	1 (2.3%)	0.122
Sugimoto stage III	12 (18.5%)	4 (19%)	8 (18.2%)	0.561
Sugimoto stage IV	53 (81.5%)	17 (81%)	36 (81.8%)	0.185
Follow-up time (month)	12 (3–36)	14 (3–33)	12 (4–36)	0.613

IP, interphalangeal joint.

### IP: interphalangeal joint

MAP and HR were greater than Ta and Tc in both groups at Tb, but the differences were not significant. The three time points corresponding to life symptoms are detailed in Table 2.

**Table 2:** Changes in vital signs in the two groups at each time point.

Item	Ta	Tb	Tc	*p*-value
≤3 year (*n* = 21)				
HR (per min)	111.2 ± 9.8	117.3 ± 14.2	113.2 ± 9.2	0.271
RR (per min)	24.3 ± 1.8	24.9 ± 2.5	23.9 ± 2.3	0.536
MAP (mmHg)	63.1 ± 3.7	66.5 ± 7.2	61.5 ± 4.2	0.123
SpO_2_ (%)	99.2 ± 0.7	99.3 ± 0.6	99.5 ± 0.4	0.684
>3 year (*n* = 44)				
HR (per min)	112.6 ± 9.1	114.3 ± 10.4	115.2 ± 9.4	0.342
RR (per min)	24.1 ± 3.2	24.4 ± 2.1	23.9 ± 4.1	0.791
MAP (mmHg)	62.3 ± 2.7	62.5 ± 3.8	62.2 ± 3.4	0.456
SpO2 (%)	99.1 ± 0.6	99.2 ± 0.8	99.4 ± 0.6	0.868

Ta, anesthesia induction; Tb, skin incision; Tc, skin closure; HR, heart rate; RR, respiratory rate; MAP, mean arterial pressure; SpO2, percutaneous arterial oxygen saturation.

Data are expressed as numbers (%) or as medians (IQRs).

The overall success rate of UDFMNB was 93.8%, with 6.2% of PTT patients requiring conversion to general anesthesia due to block failure. The failure rate was greater in the under 3-year-old group than in the 3-year-old group (4.62% vs. 1.54%).

The operative time was 15 (14–23) min, and the total time from entering to leaving the operating room was 31 (29–41) min, with no significant differences between the two groups. No surgery-related complications were observed in any patient. In each group, one patient developed mild postoperative skin redness and swelling at the puncture site, which resolved after symptomatic treatment, with no statistically significant difference in anesthesia-related complication rates between the groups.

The data are presented as the means±SDs or medians (IQRs). At the final follow-up, all patients regained thumb mobility, and no patient required reoperation. Pain was assessed using the 11-point numeric rating scale (NRS) at different time points: intraoperatively (during skin incision), immediately postoperatively, and on postoperative Day 1. The median NRS scores were 2 (1–3) at the time of skin incision and 1 (0–1) at the other two time points, with no significant intergroup differences. Both surgeons and parents/guardians reported high levels of satisfaction with anesthesia, with median scores of 5 (4–6) and 6 (5–7), respectively, with no statistically significant differences between the groups (Table 3).

**Table 3:** UDFNB block and surgical outcomes of the patients.

Item	Total *n* = 65	≤3 year *n* = 21	>3 year *n* = 44	*p* value
Quality of the block				
Complete block (grades 1 and 2)	61 (93.8%)	19 (90.5)	52 (98.1%)	**0.003**
Incomplete block (grades 3 and 4)	4 (6.2%)	2 (9.5%)	2 (1.9%)	**0.001**
Surgery time (minutes)	15 (14–23)	16 (14–21)	14 (13–23)	0.112
Total OR time (minutes)	31 (29–41)	33 (29–38)	30 (27–41)	0.092
Satisfaction of the surgeon*	5 (4–6)	5 (4–6)	5 (4–6)	0.125
Satisfaction of the parents*	6 (5–7)	6 (5–7)	6 (5–7)	0.541
Intraoperative NRS**	2 (1–3)	2 (1–3)	2 (1–3)	0.717
Immediate postoperative NRS	1 (0–1)	1 (0–1)	1 (0–1)	0.361

*, 7-point Likert scale. **, 11-point NRS.

OR, operation room; NRS numerical rating scale.

Data are expressed as numbers (%) or as medians (IQRs). The bolded values indicate that the *P*-values < 0.05, and the differences are statistically significant.

## Discussion

Currently, few studies have focused on anesthesia protocols for A1 pulley release surgery in pediatric trigger thumbs. Although various anesthetic techniques, including general anesthesia, nerve blockade, and local infiltration, are theoretically feasible, the practical challenges associated with these methods need to be considered. Specifically, young children often demonstrate poor compliance with nerve blockade or local infiltration alone, and the long-term effects of general anesthesia on the developing brain remain unclear (11, 20). Therefore, a balanced approach incorporating moderate sedation and analgesia or relatively light general anesthesia combined with nerve blockade may represent an ideal anesthetic strategy for pediatric trigger thumb release surgery.

Liu et al. (21) demonstrated that ultrasound-guided inferior median nerve block in the forearm provides more effective analgesia, higher success rates, and lower dosages of both general and local anesthetics compared to the anatomical landmark-guided block method. This technique is suitable for pediatric patients undergoing open surgery for trigger thumb. However, it should be noted that the study focused solely on the use of ultrasound guidance for nerve block and included only patients under the age of 3. In contrast, our study divides participants into two groups based on age and employs ultrasound-guided forearm median nerve block for both groups.

This study revealed that UDFMNB is a safe and effective anesthesia technique in the southern Chinese population that can meet the requirements of thumb trigger finger release surgery in children, with a high overall success rate (93.8%) and a minimal incidence of complications. Although UDFMNB block failure requiring conversion to general anesthesia was rare (6.2%), it occurred more frequently in children under 3 years of age than in children over 3 years of age, indicating age-related technical challenges. The higher failure rate in younger children may be related to a smaller nerve diameter and decreased anatomical accessibility during needle positioning. The intraoperative and postoperative pain scores were low, and no anesthesia or surgery-related complications were observed. The high level of satisfaction reported by surgeons and parents, as well as the general recovery of thumb mobility, further support UDFMNB as a safe and feasible alternative to general anesthesia in this population.

Due to the poor cooperation of younger pediatric patients, forearm movement is more likely to occur during the block procedure, increasing the difficulty of median nerve blockade. These factors represent potential causes of block failure, thereby contributing to a higher failure rate. Although median nerve blockade carries a certain failure rate, the advantages of regional nerve blockade must be acknowledged. For instance, it avoids any airway manipulation during anesthesia, thereby reducing complications such as aspiration and postoperative sore throat. Given the variability in children’s cooperation, the choice of sedative prior to nerve blockade has not yet been standardized in our institution. For cooperative patients, intranasal dexmedetomidine was administered, whereas propofol was used via intravenous cannulation for less cooperative patients. In future studies, we will standardize the pre-block sedation protocol to enhance the reliability of research outcomes.

The traditional median nerve block (22) based on surface landmarks requires patients to cooperate and complete active wrist flexion movements to protrude the tendons of the flexor carpi radialis and palmaris longus muscles. This limits the use of this method in children, as they must undergo general anesthesia beforehand to comply with and tolerate the pain associated with the puncture. Under general anesthesia, pediatric patients are unable to perform active wrist flexion, and the thicker subcutaneous fat in children increases the difficulty of identifying the flexor carpi radialis longus and palmaris longus tendons. In recent years, the use of ultrasound-guided nerve blocks in pediatric patients has been increasing (13, 23). Under the guidance of ultrasound images, the needle tip can easily be punctured to the target nerve, and the anesthetic can be injected to surround the target nerve under image guidance (12). There was no significant difference in the increase in HR or MAP at Tb compared with Ta and Tc in either group of pediatric patients. This finding indicates that both groups achieved satisfactory results through UDFMNB.

The surgical procedure for trigger thumb A1 pulley release in children is simple and relatively quick and is often performed under local infiltration anesthesia in adults. Although studies have indicated that the wide-awake local anesthesia no tourniquet (WALANT) technique is safe and feasible for pediatric orthopedic surgeries (24), our institutional experience suggests that there are certain difficulties in implementing the WALANT technique during trigger thumb surgery in children. First, due to the young age of the patients, compliance is poor, and they are unable to tolerate the pain associated with infiltration anesthesia. Second, most surgeons believe that this can lead to tissue edema and hinder the surgical procedure. Finally, ensuring that the affected finger remains immobilized during surgery, which can affect intraoperative manipulations, is challenging.

Although brachial plexus block is considered an effective option for hand surgery, it carries risks such as pneumothorax, hemothorax, phrenic nerve palsy, and vascular injury (25). Compared with different types of proximal brachial plexus blocks, the onset time of sensory blockade in the forearm distal peripheral nerve block group is faster (12). This may be attributed to the alternating changes in the thickness of the epineurium and the nerve itself along its route, as both gradually decrease from proximal to distal. This reduction in thickness likely facilitates the deeper penetration and diffusion of local anesthetics into the nerve.

Our study demonstrates that UDFMNB can achieve favorable outcomes in PTT release surgery, and it should be noted that all included procedures were performed by senior pediatric anesthesiologists who had undergone extensive clinical training. In clinical practice, beginners must acknowledge the existence of a learning curve. All operators should receive standardized hands-on training, with particular attention given to key challenges such as needle–probe alignment, hand–eye coordination, needle insertion planning, and long-needle handling. The use of both basic and advanced simulators may help improve the learning curve for musculoskeletal ultrasound-guided procedures (26).

This study demonstrated that ultrasound-guided distal forearm peripheral nerve block can achieve favorable outcomes in pediatric trigger thumb surgery, which undoubtedly has several limitations. First, this study is a retrospective cohort study; while retrospective analysis may more accurately reflect real-world clinical practice, such designs are inherently susceptible to selection bias. Second, this was a single-center study, with data derived solely from a pediatric specialty hospital in southern China, and the sample size was limited. Finally, the absence of randomized allocation may have led to a baseline imbalance between groups, potentially affecting the accuracy of the findings. In the future, multicenter, randomized controlled trials are warranted to further validate these results.

## Conclusion

In summary, our investigation suggests that ultrasound-guided distal forearm peripheral median nerve block has high success rates and an excellent safety profile for analgesia in pediatric trigger thumb release surgery, making it suitable for clinical application and wider adoption.

## Data Availability

The original contributions presented in the study are included in the article/supplementary material, further inquiries can be directed to the corresponding authors.
